# Attentional Selection Accompanied by Eye Vergence as Revealed by Event-Related Brain Potentials

**DOI:** 10.1371/journal.pone.0167646

**Published:** 2016-12-14

**Authors:** Maria Sole Puig, Josep Marco Pallarés, Laura Perez Zapata, Laura Puigcerver, Josep Cañete, Hans Supèr

**Affiliations:** 1 Dept of Cognition, Development and Educational Psychology, University of Barcelona, Barcelona, Spain; 2 Neuroscience Inst, University of Barcelona, Barcelona, Spain; 3 Pediatric Research Inst, Hospital Sant Joan de Déu, Barcelona, Spain; 4 Mental Health Dept, Consorci Sanitari del Maresme, Mataro, Spain; 5 ICREA, Pg. Lluís Companys, Barcelona, Spain; University of Electronic Science and Technology of China, CHINA

## Abstract

Neural mechanisms of attention allow selective sensory information processing. Top-down deployment of visual-spatial attention is conveyed by cortical feedback connections from frontal regions to lower sensory areas modulating late stimulus responses. A recent study reported the occurrence of small eye vergence during orienting top-down attention. Here we assessed a possible link between vergence and attention by comparing visual event related potentials (vERPs) to a cue stimulus that induced attention to shift towards the target location to the vERPs to a no-cue stimulus that did not trigger orienting attention. The results replicate the findings of eye vergence responses during orienting attention and show that the strength and time of eye vergence coincide with the onset and strength of the vERPs when subjects oriented attention. Our findings therefore support the idea that eye vergence relates to and possibly has a role in attentional selection.

## Introduction

The attention system plays a prominent role in selecting incoming sensory information by modulating stimulus-evoked responses. Much focus is given to the role of the parieto-frontal region of the cerebral cortex in visual attention. Attention originating in these areas is carried by cortical feedback projections to other sensory areas affecting the late stimulus-evoked responses. Other brain structures being the thalamus, brainstem and cerebellum are also known to be involved in attention. It has been suggested that thalamic reticular nucleus (TRN) modulates stimulus evoked responses at the very early onset in the Lateral Geniculate Nucleus [1]. The brainstem, by means of the Superior Colliculus plays a crucial role in the process of target selection by attention [2] and the cerebellum may be critical in the maintenance of predictive activity [3].

Brain signals reflecting attention are observed in different components of the scalp event related potentials (ERPs) responses. Many studies show larger sensory evoked ERP responses for attended targets than for unattended ones [4–6]. Specifically, the late components of the ERP responses are in index of attentional shift to the cued location [7,8] and of the selection and processing of the cued stimulus [9,10].

**Fig 1:**
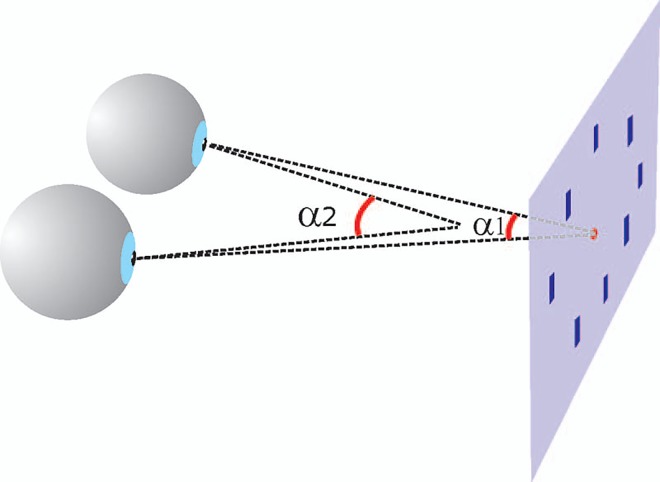
Schematic explanation of the modulation in the angle of eye vergence during a visual task. The eyes focus on a single point in space, i.e. the fixation spot. The eyes briefly converge when orienting attention to one of the eight peripheral bars. The vergence angle (α1) becomes then larger (α2).

Recently we reported a relation between eye vergence modulation and covert attention (Fig 1). We observed that the eyes briefly converge after the presentation of a stimulus that indicated the location of an upcoming visual target but not or weakly after a stimulus that was not informative about the target location [11]. Moreover, detected stimuli were accompanied by vergence responses whereas unnoticed stimuli were not. Also stimulus contrast related positively to the strength of vergence responses, and vergence responses are absent in ADHD patients [12].

The midbrain reticular formation controls eye vergence [13–18], and forms part of a broader pathway, including the frontal and parietal regions of the cerebral cortex [19–21] and cerebellum [21–23]. Besides vergence, these regions are implicated in the control of visual attention. Therefore, the aim of this paper is to provide support for the suggestion that vergence is involved in attention. We assessed whether eye vergence relates to cue evoked potentials during deployment of visuospatial attention.

## Materials and methods

### Participants

The study was approved by the Ethics committee of the Faculty of Psychology of the University of Barcelona in accordance with the ethical standards laid down in the 1954 Declaration of Helsinki. Seventeen participants took part in the EEG experiment (5 man and 12 women, 23.27±1.07 age). All participants had normal or corrected-to-normal vision. Participants received credits for courses or money for taking part in the experiment. We obtained written informed consent from all participants involved in our study.

### Apparatus

We used EventIde (Okazolab Ltd, London, UK) for the visual presentation and synchronization with the eye tracker and EEG device. The display resolution of the monitor was 1024 x 768 pixels, extending 40 x 32 degrees. The participants’ position of gaze was monitored using a binocular EyeLink II eye-tracking system at 500 Hz (SR Research System, Ontario, Canada). To compensate for head movements, we used a chinrest. Bite bars were not applied in order to exclude artifacts in the vERPs from muscle contractions of the yaw.

### Procedure

Participants sat in a dimly lit room, in front of the PC monitor at a distance of 45–50 cm. The eye tracking equipment was calibrated for each participant at the beginning of each set (standard binocular 9 point calibration). Before starting the experiments, participants could practice with some trials to get familiar with the task.

### Cue/no-cue experiment

The experiment consisted of 4 sets with 32 trials each (128 trials in total). After eye calibration, observers were required to fixate a central cross (5x5 pixels). After 300 ms, 8 peripheral bars (3x11 pixels, eccentricity of 7.5°) appeared. After 1000 ms, a 100% valid cue (a red line pointing to one of the peripheral positions, 3x13 pixels) or a no-cue (a red cross, 13x13 pixels) stimulus appeared for 100 ms in the central position. We presented an equal amount of cue and no-cue trials (50%/50%) which were randomly distributed. After an additional period of 1000 ms, one of the peripheral bars briefly (100 ms) changed its orientation (a tilt of 20° to the left or right). Participants had to respond by pressing a button as fast and accurately as possible to indicate whether the bar tilted to the left or to the right. Feedback was not given to the observers. Important, throughout the trial subjects were required to maintain central fixation.

### EEG

EEG was recorded using tin electrodes mounted in an elastic cap and located at 29 standard positions (Fp1/2, Fz, F7/8, F3/4, Fc1/2 Fc5/6, Cz, C3/4, T7/8, Cp1/2, Cp5/6, Pz, P3/4, P7/P8, Po1/2, O1/2). Bio-signals were re-referenced off-line to the mean of the activity of all electrodes. Taking the mean of the activity of all electrodes as a reference is commonly used and is widely accepted in ERP studies. However, other references, such as zero reference or REST [24,25] or other procedures such as Scalp Current Density [26], which is reference-free are alternatives for avoiding the influence of reference electrodes. The REST is in particular recommended for the spectral analysis, computation of coherence among difference electrode, and in functional connectivity studies. Vertical eye movements were monitored with an electrode at the infra-orbital ridge of the right eye. Electrode impedances were kept below 5 kOhm during all the experiment.

The electrophysiological signals were filtered with a low-pass of 0.01–50 Hz (half-amplitude cutoffs) and digitized at a rate of 250 Hz. Then data was low-pass filtered to 12 Hz. In this study we were interested in the cue and no-cue stimuli at the end of each trial. Therefore stimulus-locked ERPs to the cue or no-cue signals were averaged for epochs of 700 ms starting 100 ms prior to cue/no-cue stimulus (baseline) to 600 ms after the stimulus. Trials exceeding +/-100 μV in an EEG or EOG channel were automatically rejected from further analysis.

### Data analysis

To calculate the angle of eye vergence we transformed the HRef (X and Y coordinates of left [l] and right [r] eye), provided by the Eye Link II software, into angular units using algorithms designed to calculate 3D components (Sx,Sy,Sz) of both eye gaze vectors. Sx,y,z are the three coordinates which represent the intersection point of the lines describing the line of sight of each eye. When these lines do not intersect this represents the midpoint of the smallest distance between them. The transformation was performed taking into account the real distance of the screen to the observer (display-subject distance, DSD) and the actual inter-pupil distance (IPD) and converting them taking into account a factor of 15000 HRef/cm (which is a transformation given by the Eye Link II commercial software; section 4.4.2.2 “HREF” in EyeLink II User Manual). Sx,y,z are calculated as follow:

$$S_{x}=\frac{{\mathrm{IPD}(\mathrm{IPD}}^{2}(Y_{l}\;\text{-}\;Y_{r})(Y_{l}+Y_{r})+4\mathrm{IPD}(\text{-}X_{r}Y_{l}^{2}\;\text{-}\;X_{l}Y_{r}^{2}-(X_{r}+X_{l}){\mathrm{DSD}}^{2})+4(X_{r}^{2}(Y_{l}^{2}+{\mathrm{DSD}}^{2})\;\text{-}\;X_{l}^{2}(Y_{r}^{2}+{\mathrm{DSD}}^{2})))}{2{\left(\text{-}{2X}_{r}Y_{l}+{2X}_{l}Y_{r}+\mathrm{IPD}(Y_{l}+Y_{r})\right)}^{2}+8({\left(\mathrm{IPD}+X_{l}\;\text{-}\;X_{r}\right)}^{2}+{\left(Y_{l}\;\text{-}\;Y_{r}\right)}^{2}){\mathrm{DSD}}^{2}}$$

$$S_{y}=\frac{{2\mathrm{IPD}(Y}_{l}Y_{r}(-2X_{l}Y_{r}+{2X}_{l}Y_{r}^{}+{\mathrm{IPD}(Y}_{l}+Y_{r}^{}))+(IPD+X_{l}-X_{r})(Y_{l}^{}+Y_{r}^{}){\mathrm{DSD}}^{2})}{{\left(\text{-}{2X}_{r}Y_{l}+{2X}_{l}Y_{r}+\mathrm{IPD}(Y_{l}+Y_{r})\right)}^{2}+4({\left(\mathrm{IPD}+X_{l}\;\text{-}\;X_{r}\right)}^{2}+{\left(Y_{l}\;\text{-}\;Y_{r}\right)}^{2}){\mathrm{DSD}}^{2}}$$

$$S_{z}=\frac{\mathrm{IPD}(Y_{l}+Y_{r})(\text{-}{2X}_{r}Y_{l}+{2X}_{l}Y_{r}+{\mathrm{IPD}(Y}_{l}+Y_{r}))\mathrm{DSD}+4\mathrm{IPD}(\mathrm{IPD}+X_{l}\;\text{-}\;X_{r}){\mathrm{DSD}}^{3}}{{\left(\text{-}{2X}_{r}Y_{l}+{2X}_{l}Y_{r}+{\mathrm{IPD}(Y}_{l}+Y_{r})\right)}^{2}+4((\mathrm{IPD}+X_{l}\;\text{-}\;X_{r}{)}^{2}+{\left(Y_{l}\;\text{-}\;Y_{r}\right)}^{2}){\mathrm{DSD}}^{2}}$$

From these components we calculated the saccade amplitude by (1) azimuth (horizontal component of the eye movement), (2) latitude (vertical component of the eye movement) and the focalized distance by (3) vergence angles:

$$Azimuth\left(\alpha \right)=\arctan\left(\frac{S_{x}}{S_{z}}\right)$$ (1)

$$Elevation\left(\alpha \right)=\arctan\left(\frac{S_{y}}{\sqrt{S_{x}^{2}+S_{z}^{2}}}\right)$$ (2)

$$Vergence\left(\beta \right)=arctan\left(\frac{DIP/2}{\left\|S\right\|}\right)$$ (3)

Where ‖*S*‖ was calculated as:

$$\left\|S\right\|=\sqrt{S_{x}^{2}+S_{y}^{2}+S_{z}^{2}}$$ (4)

Only correct trials and trials without saccades (detected using an amplitude threshold) were analyzed. When the eyes were positioned outside the central fixation area (a virtual, i.e. an invisible square of 5*5 degrees centered on the fixation point) during the cue/no-cue period the trial was excluded for further analysis. Excluded trials were mainly trials with saccades and blinks. In [11] we have shown that micro-saccades do not change the results on vergence. After filtering the data in the analysis we used 49.52% (N = 823) cue and 50.48% (N = 839) no-cue trials. Total amount of trials excluded was 13.64%. Trials were excluded for both for vergence and EEG analysis. Thus exactly the same trials that were used for vergence calculations were also used for the EEG analysis. We estimated the onset latency of vergence by calculating the mean vergence of all subjects and then find the time points when the vergence responses in the cue condition start to significantly (t-test; p<0.01) differ from the vergence responses in the no-cue condition (see also 11). The first point in the series of 5 significant points was taken as the onset latency of eye vergence and was used for aligning the EEG signal.

For the ERP analysis we have applied the Maris and Oostenveld clustering method [27], which is a way to correct multiple time points comparisons in EEG/MEG data. Only those clusters showing p<0.05 corrected for multiple comparisons are represented. Point by point pair-wise comparisons between the cue and no-cue conditions was applied to reveal significant differences. In order to determine whether there was a relationship between the ERP responses and vergence, we computed the cross-correlation between them. In this analysis, correlation is performed shifting one of the signals certain milliseconds in time (lag). Formally, the cross-correlation at certain lag is computed as:

$$r(lag)=\frac{\sum \left(VERG(t)-\bar{VERG})(ERP(t+lag)-\bar{ERP}\right)}{\sqrt{\sum {\left(VERG(t)-\bar{VERG}\right)}^{2}}\sqrt{\sum {\left(ERP(t+lag)-\bar{ERP}\right)}^{2}}}$$

where ERP(t) and VERG(t) are the values of Event-Related Potentials and Vergence at time t respectively. Cross-correlation was computed at single trial level in the electrode showing maximum difference between cue and no-cue conditions with lags covering from -400 ms to 400 ms. Comparison between cue and no-cue cross-correlation values was assessed by means of the non-parametric sign-test at subject level.

## Results

**Fig 2:**
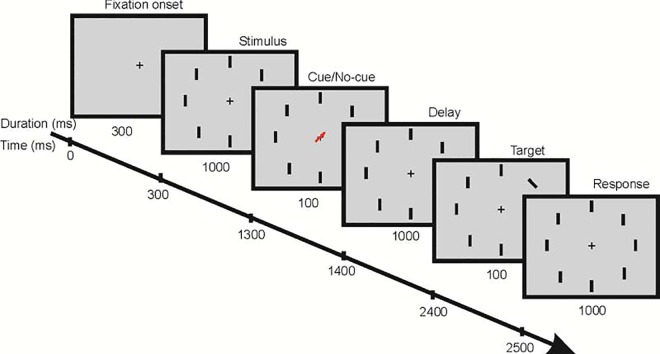
Illustration of the visual attention task.

Human subjects were tested in a cue/no-cue paradigm while eye positions and vERPs were monitored. Participants were required to fixate a central cross surrounded by 8 peripheral bars (Fig 2). One of the bars briefly changed its orientation and participants had to indicate by a button press the direction of the change in orientation. Improved behavioral performance was observed for targets in the cued condition compared to targets in the no-cue condition (mean reaction times and SEM for cued and un-cued targets: 560.3±7.3 ms vs 644.3±8.9 ms, p<0.001; percentage correctly detected cued and un-cued targets: 91.4% vs. 81.7%).

**Fig 3:**
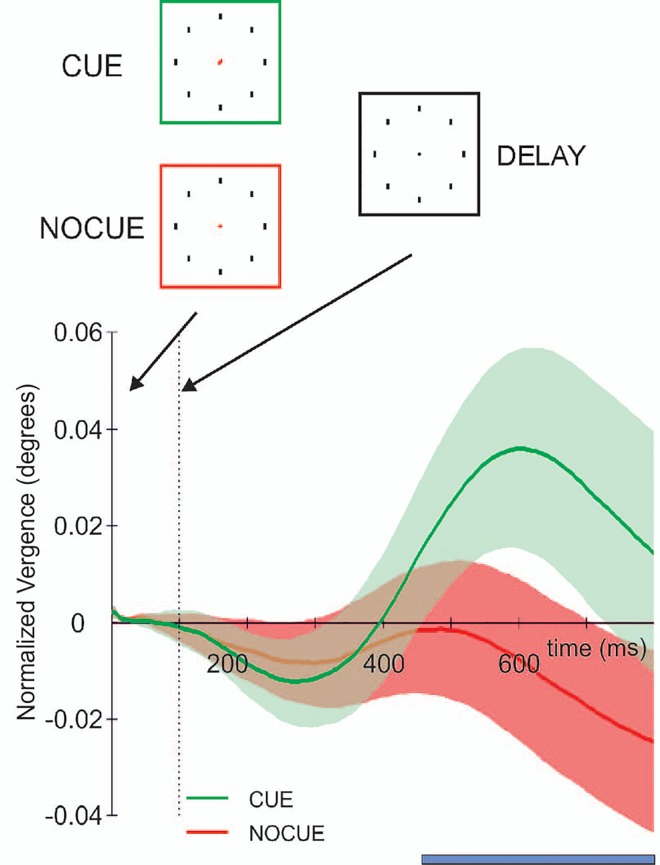
Vergence eye movements. The normalized average (across all subjects) size of the angle of eye vergence in the cue (green) and no-cue (red) conditions over time. Time points (blue) indicate a significant difference in angle of eye vergence between both conditions. Shaded areas represent ±1 times standard deviation around the mean. Time is from cue onset.

We then calculated the vergence angle. The average absolute vergence angle during the first 200 ms after cue presentation was 7.17 degrees and was identical for both cue and no-cue condition. Thereafter starting around 300 ms after the presentation of the cue we observed a change in the angle of eye vergence (Fig 3), which was not seen in the no-cue condition. This difference in vergence responses between the cue conditions became significant (p<0.01) after 466 ms.

We tested fixation stability by analyzing the variability in horizontal and vertical eye position during gaze fixation. We calculated the standard deviation (STD) of the eye position over time for both eyes separately in both conditions after the onset of the cue stimuli. In agreement with previous report [11] we found no significant (t-test; p>0.1) differences in STDs. These results are indicative of accurate gaze fixation in both cue conditions. We complemented this analysis by comparing the deviations in vergence angle between both conditions. In both conditions the STDs in vergence angle was similar (cue: 0.047; no-cue: 0.047; t-test; p = 0.89). We further assessed whether the rate and direction of micro-saccades, which is a measure of covert attention [28,29] was different in the two conditions. The rate of micro-saccades was 40.35% and 33.37% in the cue and no-cue conditions, respectively. The difference was statistically significant (chi-square; p<0.0032). No tendency was observed in the directions of micro-saccades and the distribution in directions between the cue conditions were not different (Wilcoxon rank sum test; p = 0.46).

**Fig 4:**
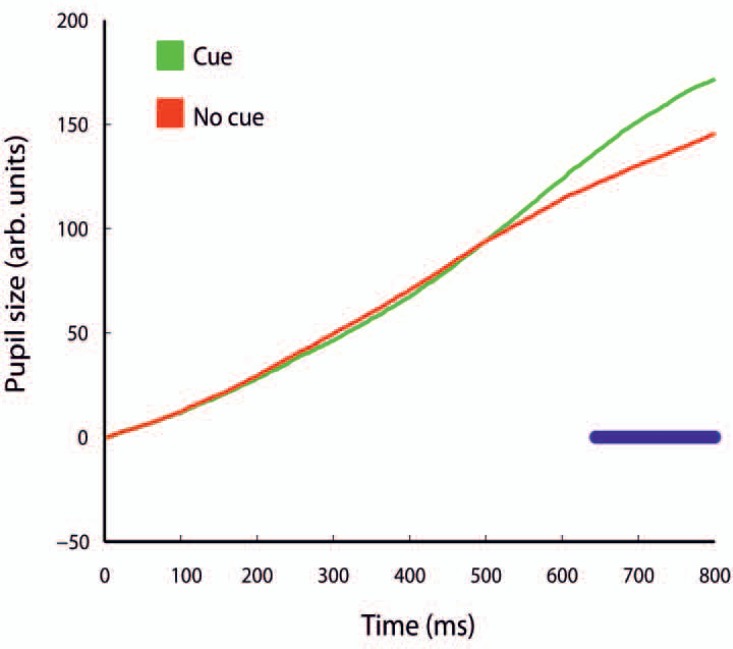
Pupil size and auditory cueing. The average pupil size in the cue (green) and no-cue (red) conditions over time. Time points (blue) indicate a significant differences between conditions.

The control of vergence and pupil size are partly correlated. We observed that for cue and no-cue conditions pupils increased steadily during the trial. In the cue condition pupil size grew larger than in no-cue condition and the difference became significant around 640ms after cue onset (Fig 4), which is around the same time when vergence responses reached a maximum (Fig 3). Thus the temporal modulation in pupil size does not match the one of vergence responses and agrees with our previous findings [11].

**Fig 5:**
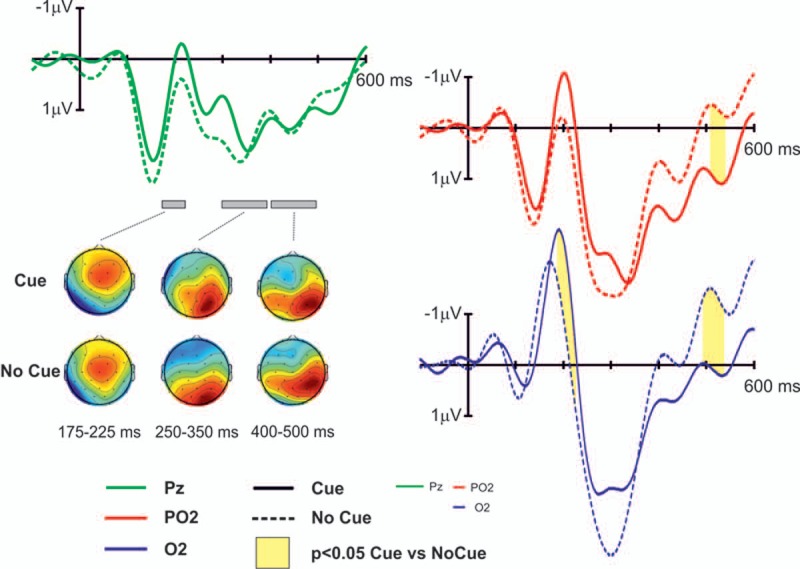
Event-Related potentials. Event-Related potentials (y-axis) associated to the cue signal (solid line) and no-cue signal (dashed line) for three parieto-occipital electrodes: O2 (blue), Pz (green) and PO2 (red). Significant differences (p<0.05 corrected for multiple conditions, see Materials and Methods sections) between cue and no-cue conditions are indicated with solid yellow areas in the corresponding electrodes. In addition, voltage maps are included at selected time windows (175–225 ms, 300–400 ms and 400–500 ms) for descriptive purposes. Time is from cue onset.

We next analyzed the vERPs responses. The two vERPs showed the classical visual N1 peaking at 190 ms in the cue condition and 170 ms in the no-cue condition, with more negative activity at occipital electrodes (Fig 5). We computed the latency of the N1 component in each subject for the two conditions. The result show that the latency of the two conditions was not significant different (t-test; p = 0.2). In addition, the two conditions showed an increase in the positivity starting around 200 ms and peaking around 300 ms with a maximum at right parieto-occipital electrodes. This late peak has previously shown to correspond to the N2pc component [30]. Point by point pair-wise comparisons between the two conditions at PO2 revealed significant (t-test; p<0.05) differences during the N2pc period (i.e. 500 and 540 ms after the presentation of the cue/no-cue stimuli) with greater positivity of the cue condition compared to the no-cue condition (Fig 5). Significant effects were also found in the O2 electrode, at 192–232 ms and 496–540 ms, a very similar time range than PO2.

**Fig 6:**
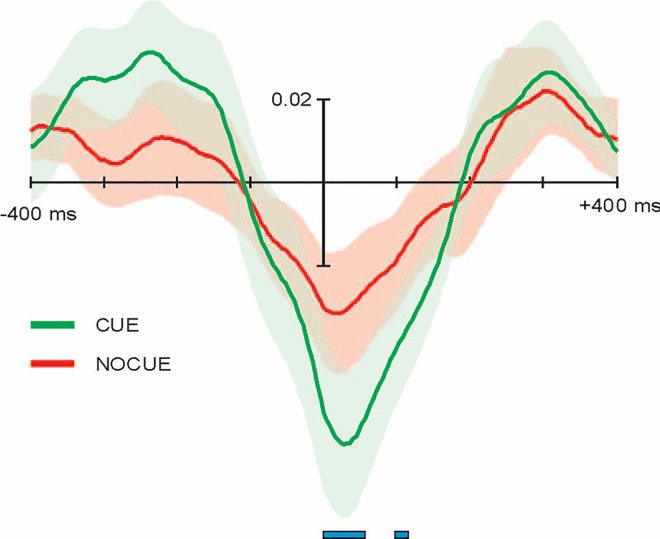
Correlation between vergence and EPRs. Cross-correlation between single-trial EEG activity and vergence at different time lags, from -400 to +400 ms in the cue (green) and no-cue (red) conditions. Solid line indicate the mean among subjects and colored areas the corresponding standard deviation of the mean. Note that the cross-correlation of the two conditions peaks around a time lag of 0 (+28 ms for the cue condition, and at +16 ms for the no-cue condition) but the significant differences between conditions (indicated by the horizontal blue lines) extend between time lags from 0 to 56 ms and from 96 ms and 116 ms.

Cross-correlation between the size of the angle of eye vergence and the single-trial EEG activity at PO2 was then assessed [31] in the cue conditions (Fig 6). This figure shows the cross-correlation with time lags going from -400 ms (EEG before eye vergence) to +400 ms (eye vergence before EEG). In the cue conditions, the cross-correlation showed a peak at +28 ms for the cue condition, and at +16 ms for the no-cue condition. The two peaks indicate the time lag where the correlation between vergence and ERPs for the two conditions present the highest negative value. This would suggest that the correlation does not have a peak at 0 (direct correlation between vergence and ERPs) but that there is a certain delay of ERPs relative to vergence. When comparing the two cross-correlation patterns, we found a significantly greater correlation of the cue condition compared to no-cue condition using sign-test between 0 and 56 ms and between 96 ms and 116 ms (Fig 6).

**Fig 7:**
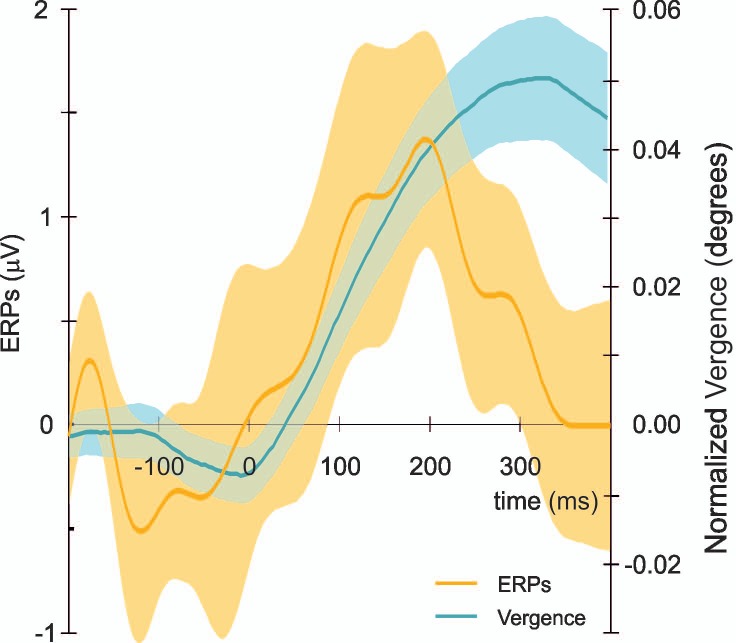
Vergence and ERPs responses. Average (across all subjects) normalized vergence (blue) and ERPs (orange) responses. ERP response is aligned to the onset of mean eye vergence across subjects. Shaded areas represent ±1 standard deviation around the mean. Time is from mean onset latency of eye vergence. Note that we plotted the ERP component with positive values up (contrary to the traditional orientation, negative up) in order to easily compare it with vergence values.

We further analyzed a possible relation between eye vergence and vERPs by aligning the VERPs to the onset latency of the vergence response for each subject. First we calculated the mean onset latency of eye vergence responses and used this value to align the single trial vERPs. If no temporal relationship exists between eye vergence and vERPs we expect to average out the increase in positivity in vERPs across trials and subjects and observe a rather flat EEG signal. However a clear peak response in the vERPs starting around the same time as the onset of the modulation in the angle of eye vergence was observed (Fig 7).

## Discussion

The present results show a correlation (both in amplitude and onset latency) between eye vergence and visual evoked potentials (vERPs) during deployment of top-down attention. Maintenance of attention is unlikely to explain the observations as both vergence and ERPs show a transient peak response. A difference in motor preparatory process neither can explain the data as in both cue and no-cue condition a response was required. Our previous study rule out a near triad effect as the angle of eye vergence does not correlate with the distance of the target to the eyes neither with pupil changes [11]. Our current data show that the eyes converge while pupils dilate. This is contrary to the near triad effect. Disparity is an alternative explanation for the observed vergence responses. However, several arguments argue against an explanation by disparity. Vergence responses to visual targets were also seen in an auditory cueing paradigm indicating that differences in visual stimulus attributes were not causing the enhanced vergence responses [11]. Similarly the strength of vergence response predicted the behavioral responses to otherwise identical visual stimuli, i.e. fixation cross and peripheral target [11]. Cueing is only effective when the target is presented at the moment when vergence responses occur [11]. Together these results indicate that vergence responses relate more to cognitive processing of visual information than to differences in fixation disparity. An idea supported by the observation that the strength of vergence responses are associated to cognitive style [32]. The magnitude of the eye vergence effect in the present study is smaller than our previous [11]. Possibly the use of a chinrest instead of a bite board may have caused some additional noise in the vergence signal. In addition, we applied slightly different filter setting for artifact removal allowing more trials to pass.

Some studies propose that the N2pc of the target induced vERPs indexes mechanisms involved in deployment of visuospatial attention [30], localizing and identifying relevant stimuli in the scene through enhancement of their features [33], filtering distractors [34], or both [35]. Stimuli presented on the midline between left and right visual fields however are known to not elicit N2pc activity [36]. Also other late target evoked ERP components (e.g. EDAN) reflect spatial attention processing [7]. The interpretation that the EDAN is an index of shifting attention to the cued location has been supported by many studies. However, a recent study proposed that the EDAN component is actually an N2pc elicited by attentional shift to the cue stimulus rather than to the cued location [9,10]. In most of these studies, late attention related EEG components are responses to the attended target. In our study we evaluated the EEG responses evoked by the central cue/no-cue stimulus as we were interested in assessing attention related vergence. In our case both cue and no-cue stimuli are attended (equally) and therefore the comparison between previous vERPs findings and our current ones should be taken with caution.

We found differences in vERPs and eye vergence between cue and no cue condition. This difference may relate to the different extent of the visual region covered by attention between cue conditions. It was observed that neural activity preceding the objects in multiple retinotopic visual areas correlated with the size of the attended region [37]. Top-down attention not only causes enhanced responses to stimuli at attended location, but also leads to synchronized neural activity in visual areas. For example, a study [38] showed how attentional selection appears to be mediated by changes in the synchrony of responses of neuronal populations, in addition to the modulation of the firing rate of individual neurons. The observed differences in eye vergence and vERPs between cue conditions could therefore reflect an increase of activity and/or synchronization in early visual areas.

Our findings replicate our previous results and support the conclusion that eye vergence is a marker of, and perhaps has a role, in covert attention [11]. Of course we cannot make any causal claim but a role of vergence in attention would be in line with the growing evidence of a role for fixational eye movements in visuospatial covert attention [28,29,39–41]. Also they concur with observations of a relationship between binocular disorders and attention problems in various mental disorders like ADHD [42]. Indeed we observed strong vergence responses in healthy children but not in ADHD children [12]. The mechanisms underlying the late evoked ERP could thus play a role concerning attentional deficits in attention-deficit/hyperactivity disorder [43]. For instance, using neuro-imaging techniques a study reported brainstem abnormalities in ADHD patients and showed 93% prediction accuracy [44], and recently we were able to classify ADHD children with high accuracy [45]. Poor binocular coordination in dyslexic children also suggest a deficiency in the visual attention processing as well as an immaturity of the ocular motor saccade and vergence systems interaction [46].

Neurons in the brainstem, in particular in the midbrain reticular formation, control eye vergence [13–18]. The reticular formation forms part of a broader pathway, including the frontal and parietal regions of the cerebral cortex [19–21] and cerebellum [21–23] that is involved in the control of vergence eye movements. These same structures that control vergence also form part of the attention system of the brain. Cortical feedback, a neural substrate of top-down attention, prominently targets cells located at the foveal region of the visual cortex [47]. Vergence, which is the opposite movement of both eyes changes binocular disparity, and may activate disparity cells at the foveal region. These cells have small receptive fields and are therefore most sensitive to small disparity changes. A possible role for vergence in attention may be to change feedback interactions necessary for shifting attention to perceive visual stimuli [48–50].

In conclusion, our current observations support a link between selective visual covert attention and eye vergence. Clearly more studies are needed but if vergence has a role in attention, it implies an oculomotor pathway in top-down attention. This idea may put current models of visual attention in a new light and may provide novel insights not only into the neurobiology of visual attention, but also into attention disorders.
